# Relevance of IgE to novel kiwi seed allergens evaluated in kiwi allergic children

**DOI:** 10.1186/2045-7022-5-S3-O24

**Published:** 2015-03-30

**Authors:** Hillevi Englund, Johanna Hidman, Otti Bengtsson-Gref, Caroline Nilsson, Peter Brostedt

**Affiliations:** 1 R&D, ImmunoDiagnostic, Thermo Fisher Scientific, Uppsala, Sweden; 2 Sach's Children's Hospital, Södersjukhuset, Stockholm, Sweden

## Background

Kiwi fruit is a common food allergen and severe allergic reactions are observed. The current diagnostic accuracy is poor with a reported diagnostic sensitivity of 65% in a large European cohort. Recently, two new kiwi allergens were registered, both being storage proteins and both isolated from kiwi seeds. Storage proteins are often linked to severe clinical reactions in other food allergies. The aim of this study was to evaluate sera from Swedish children with documented kiwi allergy, and investigate their IgE-reactivity to purified seed allergens.

## Methods

Kiwi seeds were isolated, extracted and analyzed using chromatographic methods, MALDI-TOF and SDS-PAGE. Patient samples from children with documented primary kiwi allergy were used for evaluation. Care was taken to not include patients primarily sensitized to birch pollen in this study, to avoid cross-reactivity between Bet v 1 and the homologous Act d 8. IgE-immunoreactivity towards all four purified proteins, as well as the whole kiwi seed extract, was studied.

## Results

2S albumin (Act d 13), 11S globulin (Act d 12), 7S globulin, and LTP (Act d 10) were purified from kiwi seeds and protein identities were confirmed by MALDI-TOF (Act d 10 and Act d 12), gelfiltration and SDS-PAGE. Levels of allergen-specific IgE in patients’ sera were analyzed and differentiated IgE-binding patterns to the purified storage proteins were observed.

## Conclusion

Swedish kiwi allergic children are sensitized to storage proteins isolated from kiwi seeds. Given the importance of IgE towards storage proteins in for example nuts, analyses of IgE-reactivity towards 11S, 7S and 2S from kiwi seeds have the potential to lead to new insights in kiwi allergy and possibly improve the diagnostic accuracy of kiwi allergy tests.

